# A De Novo PTEN Pathogenic Variant in a Young Girl with Sporadic Cowden Syndrome—A Case Report

**DOI:** 10.3390/pediatric17030054

**Published:** 2025-05-01

**Authors:** Paulina Gebhart, Christian Singer, Daniela Muhr, Christina Stein, Yen Y. Tan

**Affiliations:** 1 Department of OB/GYN and Comprehensive Cancer Center, Medical University of Vienna, 1090 Vienna, Austria; christian.singer@meduniwien.ac.at (C.S.); daniela.muhr@meduniwien.ac.at (D.M.); yen.tan@meduniwien.ac.at (Y.Y.T.); 2 Center for Forensic Medicine, DNA Central Laboratory, Medical University of Vienna, 1090 Vienna, Austria; labor@dna-wien.at

**Keywords:** PTEN, hereditary cancer syndrome, genetic testing, cancer screening

## Abstract

Cowden syndrome (CS) is a rare hereditary disorder characterized by benign overgrowth in various tissues and a high risk of breast and thyroid cancer. CS is closely associated with pathogenic variants (PVs) in the phosphatase and tensin homolog (*PTEN*) tumor suppressor gene. PVs in *PTEN* are usually inherited and estimates of de novo frequencies remain inconclusive. The diagnosis of *PTEN*-associated syndromes remains a challenge in clinical practice, due to patients showing seemingly unrelated symptoms. We report on the clinical management of a now 18-year-old female CS patient, who initially presented with macrosomia, motor development delay and later, lipomas on the abdominal wall. Genetic testing revealed a de novo *PTEN* PV *c.1003C>T(p.Arg335X)*. The PV was detected in leukocyte DNA of the patient. Using direct DNA sequencing, as well as NGS, the PV was not found in any of the tissues derived from immediate family members. However, the PV was detected in multiple samples representing other germ layers of the affected patient, which renders constitutional mosaicism unlikely. This case constitutes the first description of a de novo *PTEN* PV, in which constitutional mosaicism was systematically ruled out and underscores the importance of timely genetic testing of patients and their relatives. The diagnosis of a *PTEN* PV in early childhood allows for the implementation of a comprehensive, lifelong care plan that addresses both pediatric and adult medical needs as well as cancer risk surveillance and family planning. This not only accounts for the affected patients, but also their close family members who might be susceptible to the same PV.

## 1. Introduction

Phosphatase and tensin homolog (*PTEN*) is a tumor suppressor gene that downregulates the PI3K/AKT pathway, causing cell cycle arrest and apoptosis. Additionally, it plays an important role in the suppression of cell migration and spreading [1]. Its dysfunction results in deregulation of multiple pathways reported in human cancer. *PTEN* pathogenic variants (PVs) are inherited in an autosomal dominant pattern and can cause a wide spectrum of seemingly unrelated phenotypes, ranging from autism to cancer [2]. This wide spectrum of disorders is termed *PTEN* hamartoma tumor syndrome (PHTS), which includes Cowden syndrome (CS), Bannayan–Riley–Ruvalcaba syndrome (BRRS), *PTEN*-related Proteus-like syndrome, adult Lhermitte–Duclos disease (LDD), and autism spectrum disorders with macrocephaly [3].

Cowden syndrome (CS) is a rare hereditary condition, characterized by benign overgrowth of tissues originating from all three embryonic layers [2]. Importantly, this syndrome also leads to an increased risk of various cancers, particularly breast (85%), thyroid (35%), renal cell (33%), and endometrial cancer (28%) [4,5]. CS is estimated to affect 1:200,000 individuals [6]. However, this statistic is likely an underestimation, due to the variability of clinical features in CS patients [7]. Both familial and apparently sporadic cases have been reported in older studies. All nine exons of *PTEN* have been reported to contain pathogenic germline variants. These include missense, nonsense, and splice site variants, as well as deletions and insertions [8]. Most of these variants are inherited, and only 10–44% of patients are estimated to harbor de novo *PTEN* PVs [9]. Since its discovery in 1997 [10], a large number of novel *PTEN* variants have been reported and classified based on pathogenicity [11].

To this day, studies have failed to show a significant correlation between specific *PTEN* PVs and different types of cancers or non-malignant clinical features [12,13]. However, there seems to be a trend for early-onset disease in patients with missense variants, while those with later-onset disease tend to harbor truncating variants [13]. Additionally, truncating variants have shown to be associated with a 2–3 times higher risk for breast cancer compared to missense variants. The underlying mechanisms of these effects remain unclear [14].

Due to their rarity and variable clinical manifestations, the diagnosis of *PTEN*-associated syndromes is challenging in clinical practice. Additionally, the de novo frequency of *PTEN* PVs has been estimated at up to 46.7%, not accounting for cases that may result from mosaicism [9]. These cases typically present with a lack of family history, further complicating diagnosis. The proper diagnosis and management of *PTEN*-associated syndromes requires a thorough understanding of the complex diagnostic criteria as well as a comprehensive approach to genetic testing. This not only provides appropriate risk management for the affected patients, but also their family members.

Here, we describe the diagnosis and clinical management of a young girl with sporadic Cowden syndrome caused by a de novo PV in *PTEN*. This case describes a comprehensive approach for the clinical diagnosis and genetic investigation of an individual with a PV in *PTEN*, providing guidance on diagnostic strategies, treatment, and potential complications to watch for.

This case report was written according to the CARE Guidelines.

## 2. Case Presentation

An 18-year-old female patient with Cowden syndrome was referred to our department for clinical management.

### 2.1. Early Development

The patient (known as proband from here onwards) had been born to nonconsanguineous, Caucasian parents. She has an older sister, who is healthy. The proband had been diagnosed with macrocephaly and macrosomia prenatally. Besides maternal gestational diabetes, there were no other abnormalities during the course of pregnancy. She was born early at 36 weeks of gestation due to preterm labor, with a birthweight of 3960 g (>97 pct), body length of 52 cm (>97 pct), and a head circumference of 38 cm (>97 pct). After an initially unremarkable neonatal period, hypotonia and motor developmental delays were observed. At first, a metabolic disorder was suspected by the treating pediatricians, but metabolic screening as well as head ultrasound did not show any additional abnormalities. Abdominal ultrasound revealed hepatomegaly and bilateral nephromegaly. The proband continued to grow in a disproportional manner, with a head circumference above the 97th percentile at a body height and weight at the 75th percentile at the age of 17 months. At this age, the first MRI of the head was performed, which showed enlarged perivascular spaces. In subsequent MRI, no changes were observed regarding the constellation of this finding.

### 2.2. Genetic Testing

At the age of three, the proband began to develop lipomas on the abdominal wall. In a synopsis with the preceding medical history, the treating pediatricians suspected Cowden syndrome to be the underlying cause of these findings. This prompted genetic examination of the *PTEN* gene. The DNA samples were sent to the University Hospital Bonn for genetic testing, which was performed using the proband’s leukocyte DNA. The diagnosis was confirmed after direct DNA sequencing of *PTEN* revealed a heterozygous nonsense PV *c.1003C>T(p.Arg335X)* in exon 8. This kind of variant causes a change in sequence that brings about a stop codon instead of a codon specifying an amino acid and thus results in a truncated shorter protein that is non-functional.

Even though both parents had a negative family history, predictive testing was offered to the proband’s immediate family, as Cowden syndrome is an autosomal dominant inherited disorder and late onset may occur in a heterozygous parent [15]. The sister and both parents underwent genetic testing with direct DNA sequencing of exon 8 and the adjacent intron regions of the *PTEN* gene. Neither of the tested family members were shown to carry the PV that had been identified in the proband.

Nevertheless, even if the family members seem unaffected, there still remains a residual risk of parental germline mosaicism [15]. Therefore, after the proband was referred to our department, further genetic counseling and testing was conducted to rule out the possibility of constitutional mosaicism. For this purpose, the following samples from all three germ layers were obtained from the proband as well as her parents and sister: (1) ectodermal tissue (skin) was represented by hair follicles, (2) entodermal tissue (urogenital tract) was represented by cytospin from cells released into the urine and from vaginal swab-derived cells, and (3) mesodermal tissue was represented by white blood cells. Genomic DNA was isolated using the QIAsymphony DSP DNA Midi Kit (#937255, Qiagen) (QIAGEN Strasse 1, 40724 Hilden, Germany) on the QIAsymphony SP instrument, following the manufacturer’s protocol. Direct DNA sequencing (Sanger Sequencing) of the *PTEN* gene on chromosome 10q23 was performed on all available samples. Additionally, next-generation sequencing (NGS) was performed on the blood samples using the Illumina TruSight Cancer panel on the MiniSeq system (Illumina, San Diego, CA, USA). Read alignment, variant calling, and annotation were performed using the Sophia Genetics DDM™ platform.

In the proband, the presence of the pathogenic *PTEN* variant *c.1003C>T(p.Arg335X)* was detected in each of the obtained tissues (Table 1). However, neither parent nor sister had this variant (confirmed with direct DNA sequencing and NGS) in any of the obtained tissues.

The absence of the pathogenic germline *c.1003C>T PTEN* variant in both parents indicated either a de novo origin of the alteration or non-paternity. The latter event was ruled out by paternity testing, confirming that the genetic alteration in the proband is indeed a de novo PV.

### 2.3. Clinical Course and Management

Since the molecular genetic confirmation of the *PTEN* PV at age three, the proband received multidisciplinary care for Cowden syndrome. The lipomas in the umbilical and inguinal area were eventually resected.

At the age of twelve, the proband developed Hashimoto’s thyroiditis. Around that time, she also began to express intermittent pain in the right ankle and calf. Ultrasound and MRI revealed an arteriovenous malformation of the distal calf. After other vascular malformations were ruled out, a watch-and-wait strategy was adopted. However, the proband soon began to express increased pain in the right foot, which led to the diagnosis of a deep venous thrombosis of the right calf. While the thrombosis was mainly attributed to the compromised venous drainage, thrombophilia testing revealed activated protein C resistance due to a Factor V Leiden PV. The proband was treated with oral anticoagulants followed by sclerotherapy of the malformation.

At age 14, the proband began to notice lumps in both breasts during self-examination. She was referred to our Breast Health Center for diagnostic evaluation. Ultrasound showed multiple non-suspect hypoechogenic lesions in both breasts. Breast MRI and histopathological examination of tissue biopsies confirmed the suspected diagnosis of multiple fibroadenomas. A year later the proband began to show bloody nipple discharge of the right breast. Subsequent blood laboratory tests revealed elevated prolactin, which was followed up by MRI examination of the brain. MRI showed a small cyst-like lesion in the sellar region, suggesting the presence of a microprolactinoma. Due to the small size of the lesion and the lack of any additional symptoms, an observational approach with periodic monitoring of the blood tests and MRI imaging was adopted.

**Figure 1:**
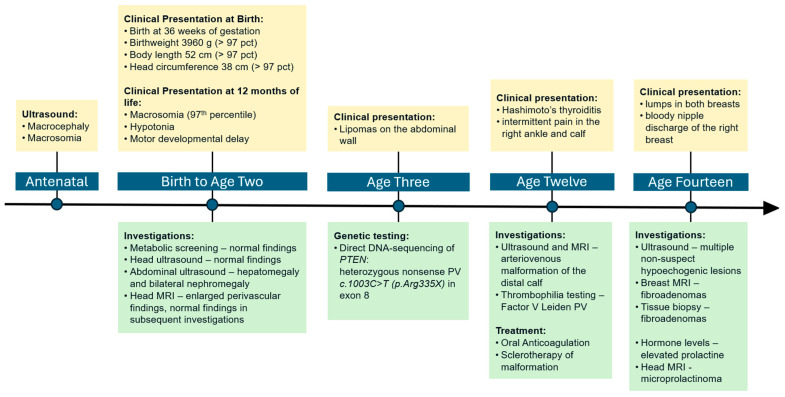
Timeline of care.

At present, the proband still receives interdisciplinary care for CS and is regularly checked for cancers at risk (breast, thyroid, kidney, and melanoma). A timeline of care is outlined in Figure 1.

## 3. Discussion

CS is an exceedingly rare autosomal dominant inherited syndrome. The definite clinical diagnosis of PHTS-associated conditions as well as recommendations for *PTEN* genetic testing are based on clinical criteria, emphasizing the importance of a thorough physical examination and complete evaluation of the patients’ medical history [3].

Like most children diagnosed with CS, our patient came to medical attention due to macrocephaly, dermatologic features (lipomas), and neurodevelopmental abnormalities (motor developmental delay) [2]. The treating pediatricians correctly recognized that these characteristics fulfilled the criteria for germline *PTEN* testing (Table 2).

The medical findings that occurred in the further clinical course (Hashimoto’s thyroiditis, vascular malformation, and fibroadenomas of the breast) additionally solidified the diagnosis [16].

Our results of DNA sequencing are somewhat limited by the missing genetic information for some of the samples, which occurred due to low DNA concentration. However, the absence of the *PTEN* PV was still confirmed in various tissues in all family members, making the presence of constitutional mosaicism very unlikely.

The *c.1003C>T;p.Arg335X* nonsense variant identified in our patient leads to a premature stop codon that terminates the translation of the protein, resulting in a loss of function. It has been reported that loss-of-function PVs in *PTEN* are more commonly linked to severe PHTS cases and the occurrence of tumors, while incomplete loss of *PTEN* function is associated with neurodevelopmental disorders [17].

Since most *PTEN* PVs are inherited, it is important to initiate predictive genetic testing of asymptomatic at-risk family members [9]. Out of the first-degree family members who were eligible for genetic testing, none were shown to carry the PV in question. Non-paternity and constitutional mosaicism were ruled out by additional genetic testing of all family members, confirming the de novo occurrence in our proband. Although we were not able to sequence samples from every germ layer from all tested family members due to low DNA concentration, the presence of constitutional mosaicism is still very unlikely.

In a prospective study by Mester et al., the frequencies of de novo *PTEN* PVs were estimated by screening clinical data and family history information of 187 families with verified PVs in *PTEN*. The frequency of de novo PVs was reported at a minimum of 10.7% in this cohort. However, the variant status of only 42 of the 187 index patients was proven to be inherited or of de novo origin by genetic testing. Additionally, no paternity testing was performed. For most participants in the study, the data were obtained from medical record documentation. Therefore, the reported de novo PV frequency may well be underestimated, since the used method did not thoroughly account for family size, non-paternity, adoption, or the presence of constitutional mosaicism [9].

The early diagnosis of PHTS not only aids in explaining a child’s distinctive external traits and developmental delay, but also helps to anticipate possible complications and therefore provide timely treatment. The most important consequence of *PTEN* PVs and the associated syndromes is the increased risk of malignancies [4,18]. However, the variable expressivity and low incidence of this syndrome makes the early diagnosis difficult. Many patients with PHTS first come to medical attention due to early onset cancers, underscoring the importance of the early diagnosis of this syndrome [2,19]. This dilemma especially concerns female patients, who are often diagnosed with PHTS later in life due to an early cancer occurrence, while male patients are more often diagnosed in childhood because of developmental delay and higher rates of macrocephaly at birth [13,20].

As soon as the diagnosis of PHTS is established, patients and their families have to be supported by a coordinated interdisciplinary care team for the surveillance and treatment of benign as well as malignant disease manifestations. The goal remains to detect malignant tumors at the earliest possible stages, to allow curative treatment. This can be achieved by following an evidence-based surveillance guideline (Table 3).

Cancer surveillance is especially important in women, as they harbor a significantly higher risk of *PTEN*-related cancers (68.4% to 86.3%) than male PV carriers (16.4% to 20.8%) [14]. This difference can mainly be attributed to the highly increased lifetime risk of developing breast cancer for women, which is comparable to *BRCA1/2* PV carriers [22]. Therefore, risk-reducing mastectomy should be discussed with all female *PTEN* PV carriers [15]. The higher possibility of developing endometrial cancer adds a further risk factor for women, while there are currently no clear recommendations for endometrial cancer screening for *PTEN* PV carriers [18]. Additionally, a study by Hendricks et al. showed higher cancer risk in sex-neutral entities like thyroid cancer in female (9.9% to 23.0%) than in male PV carriers (7.8% to 17.8%) [14].

To provide timely cancer surveillance and appropriate management of neurodevelopmental symptoms, it is crucial for clinicians to be mindful of characteristic clinical features associated with PHTS [2]. The NCCN provides regularly updated recommendations for *PTEN* genetic testing, as well as clinical diagnostic criteria for CS [15,23]. Prospective cohort studies have shown that *PTEN* PVs are found in about 30% of patients meeting the NCCN consortium diagnostic criteria [8,24].

Neither of the first-degree family members was shown to carry the PV in any of the tested tissues, and paternity testing confirmed true paternity. While the proband was correctly diagnosed early and has been provided with appropriate care and preventative measures throughout her life, living with the knowledge of a highly elevated cancer risk as well as the prospect of further risk-reducing surgery leads to emotional distress and implications for family planning. All affected PV carriers should therefore be offered psychological support and counseling for family planning as needed. As female PV carriers are reportedly diagnosed later than their male counterparts, we believe that more studies focusing on personalizing the diagnosis and surveillance of women with PHTS are needed.

## 4. Conclusions

This case report demonstrates the importance of a comprehensive clinical investigation and thorough genetic evaluation in children with features suggestive of Cowden syndrome. A de novo *PTEN* PV (*c.1003C*>*T*;*p.Arg335X*) was identified, and constitutional mosaicism was systematically ruled out through multi-tissue analysis; to our knowledge, this is the first report to do so in this context. A timely diagnosis enabled the implementation of appropriate multidisciplinary care, including cancer surveillance and management of benign manifestations. These findings reinforce the value of predictive testing and counselling for at-risk family members. Overall, this case contributes to the growing body of knowledge on PTEN-related disorders and offers practical insights for future diagnostic and clinical management strategies.

## Figures and Tables

**Table 1:** Combined results of direct DNA sequencing and NGS.

	Sample Material	*PTEN* Status
Proband	Blood	Nonsense Variant *c.1003C*>*T*(*p.Arg335X)*
	Urine	Nonsense Variant *c.1003C*>*T*(*p.Arg335X)*
	Hair	n/a *
	Vaginal Swab	Nonsense Variant *c.1003C*>*T*(*p.Arg335X)*
Mother	Blood	Wild-type
	Urine	n/a *
	Hair	n/a *
	Vaginal Swab	Wild-type
Father	Blood	Wild-type
	Urine	Wild-type
	Hair	n/a *
	Sperm	Wild-type
Sister	Blood	Wild-type
	Urine	Wild-type
	Hair	Wild-type
	Vaginal Swab	Wild-type

* n/a = not available due to low DNA concentration. Proband = the first person in the family to be identified as having a genetic disorder and received genetic counseling or testing.

**Table 2:** *PTEN* genetic testing criteria for pediatric patients [8].

Major Criterion (Required)	Secondary Criteria
Macrocephaly (≥2 standard deviations)	At least one of the following criteria:∙ Autism spectrum disorder or developmental delay Dermatologic features (lipomas, penile freckling, trichilemmomas, oral papillomas) Vascular features (arteriovenous malformations, hemangiomas) Gastrointestinal polyps
Pediatric-onset thyroid cancer and germ cell tumors are known to be associated with CS and should therefore also prompt consideration of *PTEN* genetic testing.	

**Table 3:** Cancer surveillance recommendations for PHTS-related malignancies [18,21].

Cancer Type	Risk Estimate	Surveillance	Starting Age (Years)	Frequency
Breast	85%	Self-examination	18	
		Clinical examination	25	Annual
		MRI	30	Annual
		Mammography	40	Every 2 years
Thyroid	35%	Ultrasound	12	Every 3 years
				Annual after age 18
Renal	33%	Ultrasound	40	Every 2 years
Colorectal	16%	Baseline colonoscopy	35–40	As required ^a^
Melanoma	5%	Skin examination	18 ^b^	Annual
Endometrial	28%	Not recommended ^c^		

^a^ Consider further surveillance as required by the gastroenterologist. ^b^ Consider earlier start of screening as required by the dermatologist. ^c^ Consider ultrasound-screening as required by the gynecologist.

## Data Availability

The original contributions presented in this study are included in the article. Further inquiries can be directed to the author.
